# Comparison of survival time between two types of orthodontic fixed retainer: a prospective randomized clinical trial

**DOI:** 10.1186/2196-1042-14-25

**Published:** 2013-09-11

**Authors:** Parisa Salehi, Hooman Zarif Najafi, Seyyed Mehdi Roeinpeikar

**Affiliations:** Orthodontics Research Center, Department of Orthodontics, School of Dentistry, Shiraz University of Medical Sciences, Shiraz, Iran

## Abstract

**Background:**

The aim of this prospective clinical study was to compare the mean durability and the failure rates of two types of orthodontic retainers.

**Methods:**

Orthodontic patients (142) aged between 14 and 28 years were recruited in this study. The polyethylene woven ribbon (Ribbond, Seattle, WA, USA) retainer was compared with a 0.0175-in flexible spiral wire (Respond, Ormco, Glendora, CA, USA) retainer. When treatment was completed, the retainers were bonded from canine to canine in the maxillary and the mandibular arches of the participants. In the follow-up visits, the patients were re-evaluated every 3 months over a period of 18 months. The time taken for the retainers to remain without any fracture was appraised. Kaplan-Meier analysis and the logrank test were employed to identify significant differences in the survival functions between the groups. The rates of the retainers' failure between the groups were analyzed using Chi-square test.

**Results:**

It was revealed that the mean survival of the flexible spiral wire retainer was 15.34 ± 0.47 and 15.60 ± 0.42 months in the maxillary and mandibular arches, respectively. The mean survival of the ribbon retainer was 13.95 ± 0.55 and 14.26 ± 0.57 months in the maxillary and mandibular arches, respectively. Ribbon retainers showed a failure rate of 50% in the maxillary and 42.6% in the mandibular arches. Flexible spiral retainers showed a failure rate of 36.5% in the maxillary and 37.8% in the mandibular arches. The differences were not statistically significant. Regarding the evaluation period, the differences had limited clinical significance.

**Conclusion:**

The mean survival time and the failure rates of the polyethylene woven ribbon retainer were comparable to the flexible spiral wire retainer during the 18 months after orthodontic treatment.

## Background

The stability of the results yielded from an orthodontic treatment is always an imperative issue. Longitudinal studies evaluated post-treatment records and revealed remarkable relapses in some occlusal traits, especially in the alignment of the anterior teeth of mandible [1–4]. This verdict has made many orthodontists to believe that the only way to maintain the ideal alignment after orthodontic treatment would be a form of permanent retention. This can be a fixed retainer left in the mouth for a long period of time [5–7]. Fixed lingual retainers were introduced in the 1970s by Knelrim [8]. They were bonded to the mandibular anterior teeth and were established as a part of orthodontic treatment to prevent the relapse of the mandibular incisors.

The first generation of fixed retainers was stainless steel round wire with large-diameter section (0.030 to 0.032 in) which was bonded on the lingual surface of the mandibular canines. Later, smaller diameter braided or coaxial round wires were introduced. They had various compositions and resilience and were bonded to the all mandibular anterior teeth [9–11]. More recently, fiber reinforced composite (FRC) materials were introduced as fixed retainers [12, 13].

Fixed retainers have some particular advantages over the removable ones. They are invisible, well-tolerated by the patients and compliance-free [9]. On the other hand, fixed retainers have some disadvantages like difficulties in the retainer placing [10] and the possibility of tooth movement due to the distortion or lack of wire passivity [14]. Bonding failures [9] and wire fractures [10] are other significant problems. Bonding failure may occur either in the adhesive-enamel or in the wire-composite interface [9]. In a review study, the failure rates of fixed retainers have been reported ranging from 10.3% to 47.0% [15].

Subsequently, FRC were used as an alternative to stainless steel arch wire to reduce the bulk of the lingual retainer [12, 16]; plasma-treated, polyethylene FRC was suggested for fixed lingual retention by *in vitro* studies and case reports [17, 18]. One of the advantages of the ribbon retainers is their easy adaptation to the lingual surface and to the dental arch contours. So, they are virtually flexible, translucent, and can be considered as an excellent esthetic material which can be cured mutually with light-cured composites [19]. High biocompatibility is another clinical advantage of FRC materials. Because it contains no nickel, it can be used for the patients who are allergic to the nickel ions. Another advantage of this ribbon is that the complete breakage of the retainer does not occur frequently [20], and it can be easily repaired [19]. However, its main disadvantage is producing a rigid splint that limits the physiologic tooth movement which may contribute to a higher failure rate [15]. Although clinical follow-up studies of these fixed retainers are few, direct-bonded multi-stranded wire retainers have proved their reliability in retention of the mandibular anterior teeth after orthodontic treatments [9, 10].

Rose et al. [20] compared the reliability of two types of canine-to-canine fixed retainer in the mandibular arch after orthodontic treatment in a 24-month period. In this study, the ribbon-reinforced retainer remained in place for about 11.5 months, while the multi-stranded wire remained for about 23.6 months; this difference was considered statistically significant. In a clinical study, Dahl and Zachrisson [21] reported higher failure rates in the maxillary arch compared to the mandibular arch when they employed multi-stranded wire as a fixed retainer.

In an *in vitro* study, Foek et al. [22] compared the bond strength of the stainless steel wire retainer with various FRC retainers. They reported the stainless steel orthodontic bonded retainers had higher bond strength than FRC.

Apparently, in the patients with failed retainers, a greater increase in incisor irregularity could be measured [23]; therefore, the best method for long-term fixed retention should be selected. To the best of our knowledge, few clinical studies have evaluated the reliability of FRC retainers prospectively [20, 22, 24]. This prospective randomized clinical study aimed to compare the mean survival time and the failure rate of two fixing methods: the flexible spiral wire retainer and the ribbon reinforced retainer. In this study, they were bonded from canine to canine in the maxillary and mandibular arches of 142 patients recruited after orthodontic treatment.

## Methods

Patients (142) aged 14 to 28 years were included in this study. They were previously treated in the clinical office of the first author by using standard edgewise fixed appliances. The sample size was calculated for each group as *n* = 35, based on alpha significance level of 0.05 and power of 90%. This sampling was computed according to the study of Tacken et al. [24] who showed that 49% of the glass fiber retainers and 88% of the multi-strand retainers were still intact at the end of the study. However, considering the differences between follow-up period (18 months in our study vs. 24 months in the study of Tacken et al. [24]) and the probability that patients might drop out of the study, we considered a larger sample size.

We included the patients who had good oral hygiene, healthy periodontal condition, and no previous bonded retainer. Patients were excluded if they had deep overbite and traumatic parafunctional habits such as bruxism and clenching. Probing pocket depth and radiological examination were used to detect any periodontal problem. Patients with widespread probing depths more than 3 mm and radiographic evidence of periodontal bone loss were excluded. Patient recruitment lasted from December 2009 to August 2010. The patients were first informed verbally about the purpose of the study and then routine informed consent forms were signed either by themselves or by their parents. This study was approved by the ethical committee of the Orthodontic Research Center of Shiraz University of Medical Sciences.

Two types of fixed retainers were used in this study. Polyethylene woven ribbon (Ribbond, Seattle, WA, USA) which is a biocompatible esthetic material made from a high strength polyethylene fiber [19] and 0.0175-in flexible spiral wire (Respond, Ormco, Glendora, CA, USA). The participants were enrolled based on the inclusion and exclusion criteria, and using random allocation sequence. Random allocation was accomplished using a random number table. Polyethylene woven ribbon was used for 68 patients (29 males and 39 females), and flexible spiral wire for 74 patients (30 males and 44 females). After randomization, Chi-square test confirmed that gender distribution was balanced between the groups (*P* = 0.799). The mean age of the patients in the polyethylene woven ribbon retainer group was 18.1 ± 5.23 years, and in the flexible spiral wire retainer group was 18.2 ± 4.81 years, showing no significant difference (*P* = 0.906). Randomly assigned to the patients, the retainers were bonded to each tooth from canine to canine. The bonding of the fixed retainers was accomplished by one clinician using the routine bonding methods [20, 24].

Multi-stranded wire retainers were fabricated from a 0.0175-in multi-stranded stainless steel wire and were bonded to the lingual surfaces of the anterior teeth. Before bonding, the retainer wires were carefully shaped on the working model cast, using three-pronged wire-bending pliers (3M Unitek, Monrovia, California) to provide an exact adaptation to the lingual surfaces. The anterior segment was isolated by rubber dam. The enamel was cleaned and polished with a low-speed hand piece using rubber cap with non-fluoridated pumice for 20 s. Then, it was etched with 37% phosphoric acid gel (3M Unitek) for 30 s and washed for 20 s and air dried. A segment of 0.0175-in multi-stranded wire and Heliosit® orthodontic resin composite (Vivadent, Schaan, Liechtenstein) was used in this group. The adhesive bis-GMA sealant (Fluoro Bond, Ormco) was applied on all teeth and light-cured with a light-emitting diode (Ortholux; 3M Unitek) for 5 s per tooth. Subsequently, the multi-stranded wire was placed on the teeth, and the Heliosit® Orthodontic resin composite was applied to attach the wire to the anterior teeth over the sealant. Each tooth was light-cured for 10 s after adjusting the retainer correctly. During the setting process, the retainer wire was fixed to the inter-proximal contacts and bonded passively to each anterior tooth. After light curing, the resin composite was contoured and polished.

For bonding of the polyethylene woven ribbon, the ribbon retainer was fabricated according to the manufacturer's instruction. The anterior segment was isolated by rubber dam. All the fibers were cut to the appropriate length, using a pair of special scissors (Ribbond fiber cutter, Ribbond; Seattle).This has been practiced in the laboratory by using dental floss on the plaster casts. The ribbon was pretreated with adhesive bis-GMA sealant (Fluoro Bond, Ormco), and the excess sealant was removed using lint-free, non-cotton gauze. Tooth conditioning and isolation, and bonding agent application and curing were carried out in the same way described for the multi-stranded retainers. The adhesive bis-GMA sealant (Fluoro Bond, Ormco) was applied on all teeth and light-cured with the light-emitting diode for 5 s per tooth, followed by the application of the Heliosit® orthodontic resin composite over the sealant. The ribbon was loosely adapted to the lingual surface of the anterior teeth and then bonded directly to each individual tooth passively. This was followed by the application of a thin layer of the Heliosit® orthodontic resin composite covering the fibers. Each tooth was light-cured for 10 s after adjusting the retainer correctly.

The patients were re-examined over an 18-month follow-up period. During this period, the patients were checked every 3 months. The data reported are based on the last clinical visit in which the retainer was held without fractures. The fractures were considered in either the wire or composite, with or without a partial or total loosening of the retainer from the teeth. The interval time between bonding and loss of a retainer was measured in months. The researcher could not be blinded successfully during the failure assessment, but the statistician was blinded about the groups.

Statistical analysis was done using the Statistical Package for Social Sciences (Version 15.0, SPSS Inc., Chicago, Illinois, USA). Descriptive statistics were deliberated for the test groups. The maxillary and mandibular retainers were analyzed separately. The survival rates of the retainers were estimated using the Kaplan-Meier analysis; to identify the significant differences in the survival functions among the groups, the logrank test was used. The rates of the retainers' failure between the groups were analyzed using Chi-square test. Significance level for all statistical tests was predetermined at 0.05.

## Results

**Figure 1:**
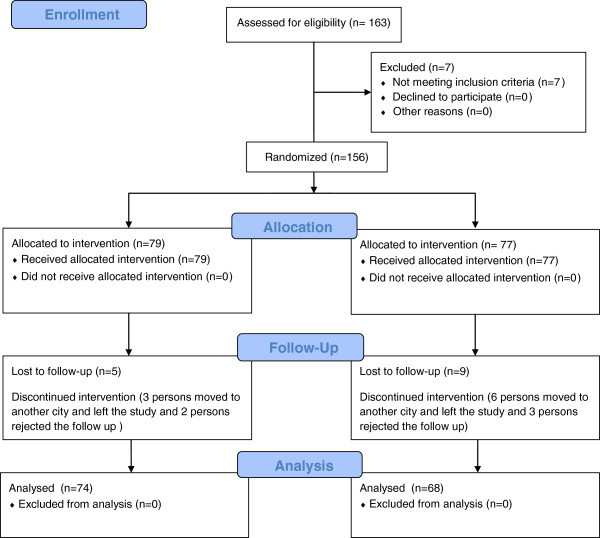
**CONSORT flow diagram.**

**Figure 2:**
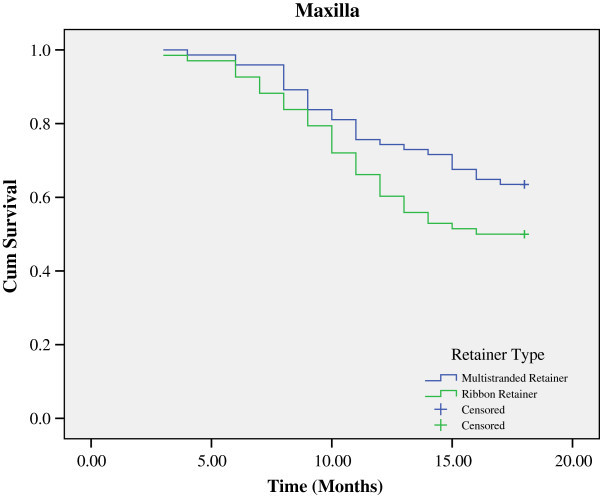
**Kaplan-Meier survival plot of the multi-stranded and ribbon retainer in the maxillary arch.**

**Figure 3:**
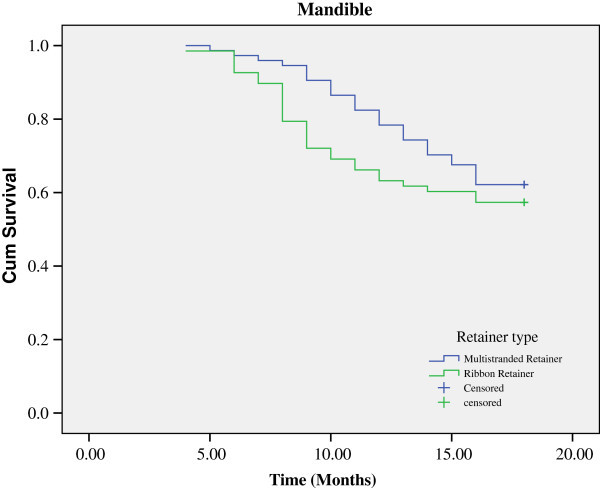
**Kaplan-Meier survival plot of the multi-stranded and ribbon retainer in the mandibular arch.**

The flow diagram reported in Figure 1 illustrates the design and conduct of the current randomized clinical trial. Descriptive statistics for the survival time in both retainer types are shown in Table 1. Figures 2 and 3 illustrate the Kaplan-Meier survival curves for the two types of retainers in the maxillary and mandibular arches. The logrank test revealed no statistically significant difference between the two types of retainers in the maxillary (*P* = 0.084) or the mandibular arches (*P* = 0.314) in terms of survival function.

**Table 1:** **Descriptive statistics for survival time (month) in the study groups**

Groups	Mean ± SE	SD	Min	Max	95% Confidence interval
Multi-stranded retainers (maxilla)	15.34 ± 0.47	4.04	4	18	14.41-16.26
Multi-stranded retainers (mandible)	15.61 ± 0.42	3.61	5	18	14.79-16.43
Ribbon retainer (maxilla)	13.96 ± 0.55	4.53	3	18	12.87-15.04
Ribbon retainer (mandible)	14.26 ± 0.57	4.70	4	18	13.14-15.39

Of the 74 multi-stranded retainers placed in both arches, 27 (36.5%) were failed in the maxillary arch during the study, while 28 (37.8%) in the mandibular arch (*P* = 0.865). Of the 68 ribbon retainers placed in both arches, 34 (50%) were failed in the maxillary arch during the study, while 29 (42.6%) in the mandibular arch (*P* = 0.390) Failure rates, both in the maxillary arch (*P* = 0.104) and mandibular arch (*P* = 0.559), were higher with the ribbon retainers compared to those of the multi-stranded retainers, although not statistically significant.

One-tooth failure was the most frequent failure of the two retainer types. Among all the retainers in all experimental groups, only in one case the multi-stranded retainer was completely detached in the maxilla. The most frequent type of failure in the multi-stranded group was retainer loosening, both in the maxilla (22/27 (81.48%)) and in the mandible (27/28 (96.42%)). In the ribbon retainer group, the most frequent type of failure was retainer fracture in the maxilla (30/34 (88.23%)) and retainer loosening in the mandible (19/29 (65.51%)). According to the survival times and the failure rates, it is possible that the differences have small clinical significance.

## Discussion

Splinting the teeth after orthodontic treatment is a common clinical procedure [19]. The main indications for the fixed orthodontic retainer are to maintain the mandibular incisor position during late growth and to maintain space closure, either after tooth extraction or after closure of diastema [20]. Multi-stranded retainers are widely used as a standard treatment option in modern orthodontics, and its retentive efficiency and reliability have been proved [9, 10]. Although traditional methods are successful, splinting the teeth with reinforcement fibers embedded in the dental composites has gained popularity [19].

In this study, newer retentive preference was compared with the standard method in the maxillary and the mandibular arches. The results showed that the reliability with the multi-stranded wire retainer was comparable to the ribbon retainer in both arches. Also, it was depicted that the mean survival time of the retainers in the mandibular arch was longer than that of the maxillary arch, regardless of the retainer type, though these differences were not statistically significant. In the 2-year study of Rose et al. [20], the ribbon-reinforced retainer remained in place for an average of 11.5 months, and this time was 23.6 months for the multi-stranded retainer. This study was conducted only in the mandibular arch. Moreover, in the study of Tacken et al. [24], the maxillary incisors and the six mandibular anterior teeth were fixed. In this *in vivo* study, glass fiber reinforced retainers showed more failure rates compared with multi-stranded retainers.

In the *in vitro* study by Foek et al. [22], the bond strengths of the different types of fixed retainers bonded to the mandibular teeth were evaluated. In this study, the stainless steel wire retainers showed higher bond strength compared to the FRC retainers. Among different FRC retainers, the Ribbond type displayed the highest bond strength.

Several reasons could describe these different observations. After orthodontic treatment, the teeth have a certain degree of mobility [25], subjecting the tooth composite interface to a greater debonding stress [20]. The FRC retainers are rigid with little flexibility which leads to the higher strain levels in the inter-dental areas under loading [26]. This issue may be related to the lower reliability of the FRC retainers. Different material properties, such as thermal expansion and water absorption of polyethylene materials, may be another reason for the lower reliability of the FRC retainer [20]. The fibers are chemically treated with plasma to contain chemically reactive molecular layers. This assures interfacial adhesion between the resin and woven polyethylene fiber. In the composite fracture, water may enter empty unpolymerized spaces along the woven fiber by capillary forces which can alter the material properties [20].

In the studies conducted by Dahl and Zachrisson [21], the spiral wire was employed as a fixed retainer in the maxillary arch and showed a higher failure rate than in the mandibular arch. In our study, the ribbon retainers showed higher failure rates in the maxillary arch than in the mandibular arch; both retainers showed longer mean survival times in the mandibular arch compared with the maxillary arch. However, these differences were not statistically significant. This might be due to the occlusal factors. When placing maxillary retainers, care must be taken to ensure that the retainer is free from occlusal trauma; this reduces the likelihood of failure [15].

## Conclusion

This study showed that the mean survival time and the rates of broken or detached ribbon retainers and multi-stranded retainers are comparable. Also, it was demonstrated that both retainer types remained longer in the mandibular arch, although the differences between the survival times of the maxillary and mandibular retainers were not statistically significant. Regarding the evaluation period, the differences were of limited clinical significance. However, the risks or benefits of prolonged use of this material for retention in orthodontic patients must be evaluated in future long-term studies.

## Authors' information

PS is an Associate Professor of the Orthodontics Research Center, Department of Orthodontics, School of Dentistry, Shiraz University of Medical Sciences. HZN and SMR are Assistant Professors of the same department.
